# Supplementary material for: Brain fog in chronic pain: Protocol for a discourse analysis of social media postings
Source: PLoS One. 2024 May 8;19(5):e0302443. doi: 10.1371/journal.pone.0302443 (PMC11078377; doi:10.1371/journal.pone.0302443)
Supplement: S1 File — (DOCX) [file pone.0302443.s001.docx]

**Appendix A**

Twitter

Using the advanced function on Twitter and Facebook the following terms will be searched:

January 1st 2016-december 31st 2019

January 1st 2020-current

- #Brainfog chronic pain
- Brain fog chronic pain
- #Brainfog #chronic pain
- Brain fog #chronicpain

Facebook:

- #Brainfog chronic pain
- Brain fog chronic pain
- #Brainfog #chronic pain
- Brain fog #chronicpain
