# Supplementary material for: Brain fog in chronic pain: Protocol for a discourse analysis of social media postings
Source: PLoS One. 2024 May 8;19(5):e0302443. doi: 10.1371/journal.pone.0302443 (PMC11078377; doi:10.1371/journal.pone.0302443)
Supplement: Attachment — Submitted filename: PONE-D-23-23957_R1_Review.pdf [file pone.0302443.s003.pdf]

PLOS ONE

Brain fog in chronic pain: Protocol for a discourse analysis of social media posting

--Manuscript Draft--

**Manuscript Number:**	PONE-D-23-23957R1
**Article Type:**	Study Protocol
**Full Title:**	Brain fog in chronic pain: Protocol for a discourse<br><br>analysis of social media posting
**Short Title:**	Brain fog in chronic pain: Social media analysis
**Corresponding Author:**	Ronessa Dass<br>McMaster University Faculty of Health Sciences<br>Hamilton, CANADA
**Keywords:**	Brain fog; chronic pain; discourse analysis
**Abstract:**	Brain fog is a phenomenon that is frequently reported by persons with chronic pain. It is commonly described as difficulties with cognition including memory impairments, attentional issues, cloudiness, and more. The current medical literature demonstrates a similar cloudiness: there is no clear taxonomy or nomenclature, no well-validated evaluations and a dearth of effective interventions. To expand our understanding of this complex phenomenon, we will perform a discourse analysis to explore how brain fog is described in public posts on social media. The utilization of this methodology will generate insights regarding the societal understanding and meaning of brain fog, by sampling a generalizable and underrepresented population. It is anticipated that the results of the proposed study will 1) help healthcare professionals better understand the lived experience of chronic pain-related brain fog and 2) generate hypotheses for future research. To conclude, by incorporating innovative and contemporary methods, this proposed discourse analysis of social media sources will generate nuanced insights, bridging the gap between researchers and health care providers, and persons with lived experience.
**Order of Authors:**	Ronessa Dass
	Tara Packham
**Response to Reviewers:**	Thank you to the reviewers for their insightful feedback. We have done our best to address all comments point by point.<br><br><br>Reviewer #1: Much of the information found on social media and forums may not be based on scientific evidence, making it difficult to differentiate between anecdotal reports and verified research findings. Therefore, I am not satisfied this is a viable methodology.<br>Thank you for highlighting your concerns. We acknowledge the limitations of social media posts in our introduction and will be sure to reiterate this point once we present our findings.<br>“Though social media data may be inherently biased, and at times inaccurate, the discourse often has real life implications (14,15). Persons with chronic pain may adopt the information they see online and may be influenced to alter their beliefs about their conditions (14,15).”<br>However, our objective for this study is not to describe what has been stated in the academic literature, but rather we are interested in understanding the discourse of brain fog by persons with lived experiences. This is particularly important considering that the term brain fog was first derived through online discussions of persons with lived experiences. In our final manuscript, we will report how people describe this phenomenon. This information will be used to provide recommendations for future areas of research.<br>Reviewer #2: This manuscript requires a major review.<br>Please find below what needs to be included in the Protocol for it to be publishable.

Powered by Editorial Manager® and ProduXion Manager® from Aries Systems Corporation

	Data collection:<br>You need to list<br>• Whether you are accessing the Twitter and Facebook public stream?/How do you access Facebook data if profiles are closed/Will go to specific brain fog sites, will you become a member of these sites?/What package you are using to access Twitter stream (.i.e. python)?<br>In accordance with published ethical guidance, we have specified this information as follows:<br>In accordance the Internet Specific Ethical Questions Framework to protect data anonymity (22), searches will occur through a public search engine, therefore no private posts, pages, or groups will be searched.<br><br>We will acknowledge this approach as a limitation in the resulting manuscript, as we may indeed miss relevant material in closed Facebook groups.<br><br>• What is the anticipate data for collection period?/Data collection duration? How many days/wks/months will you be collecting data<br><br>We started data collection after submitting our protocol. Thus, data collection occurred in October 2023 within the span of one week. Data was collected within one week to ensure feasibility as new posts generate daily on social media platform. We have specified this information at the bottom of the search strategy paragraph.<br><br><br>• What are the explicit search terms are going to be used?<br><br>We have specified the search terms “#brainfog chronic pain,” “brain fog chronic pain,” “#brainfog chronic pain” and “brain fog #chronicpain”<br><br>• Will English language filters be applied?<br>No language filters will be applied, we have added this detail in the search strategy.<br><br>• Will you exclude retweets to maintain originality<br><br>Thank you for highlighting this point, we have added it to our exclusion criteria.<br><br>• Will keep information on the tweet/fb post data attribution (full-length tweet text, tweet ID, creation time, and Twitter user information)?<br><br>All extracted potential identifiers will be deleted post manuscript completion, this information has been added at the end of the data extraction paragraph.<br><br>Annotation:<br>• Will you train classifiers to distinguish between personal and health-care related tweets/• Will you manually label all tweets?/ Will you use the content of the tweet's full text for classification or just part of?/Will you preprocess the text of the tweets by normalizing all URLs to one consistent string, removing special characters and English part of speech, converting all of the text to lowercase, and lemmatization to remove noise?/Will you look at the top 1000 occurring terms (excluding common English stop words) and manually checked if the terms were relevant to health; wellness; diseases; side effects; conditions; body parts; and/or references to other substances against standard English, medical, and slang dictionaries?<br><br>We will not be using artificial intelligence for this study, thus no classifiers or preprocessing. All searching and extraction will occur manually. We have specified this detail throughout the protocol.<br><br>Analysis:<br>• What tool will you use to undertake qualitative content analysis?<br>The mapping analysis will occur on excel and the concept analysis will be done using the qualitative software, Quirkos. This detail has been added in the data analysis paragraph on page 4.<br><br>• For transparency and rigor in qualitative research, it's conventional and widely

	recommended to have two or more individuals independently analyse the data. This practice serves multiple valuable purposes: Minimizing Bias: When two or more researchers independently analyse the same qualitative data, it helps mitigate individual biases. Each analyst brings their unique perspective and experiences, and by having multiple analysts, you reduce the risk of one person's biases significantly influencing the interpretation of the data. Enhancing Reliability: Independent analysis by multiple individuals improves the reliability of the findings. It allows for the assessment of inter-coder reliability or inter-rater reliability, which measures the degree of agreement among coders or analysts. A high level of agreement suggests greater confidence in the validity of the findings. Quality Assurance: The collaborative approach helps in identifying and resolving discrepancies or disagreements in coding and interpretation. This iterative process can lead to more robust and well-supported findings. Richer Insights: Different analysts may notice unique patterns, themes, or nuances within the data. Multiple perspectives can lead to a deeper and more comprehensive understanding of the qualitative material. Enhancing Credibility: In qualitative research, demonstrating rigor and transparency is crucial for establishing credibility and trustworthiness. Engaging multiple analysts and documenting their consensus-building process can enhance the credibility of your research findings. You need to include all the above in your protocol.<br><br>Thank you for highlighting this! We have stated this at the end of the data analysis paragraph:<br><br>To minimize bias and improve the reliability, credibility, and quality of findings data will be independently analyzed by two researchers.
**Additional Information:**	
**Question**	**Response**
**Financial Disclosure**<br><br>Enter a financial disclosure statement that describes the sources of funding for the work included in this submission. Review the submission guidelines for detailed requirements. View published research articles from *PLOS ONE* for specific examples.<br><br>This statement is required for submission and **will appear in the published article** if the submission is accepted. Please make sure it is accurate.	The author(s) received no specific funding for this work.

**Unfunded studies**<br>Enter: *The author(s) received no specific funding for this work.*<br><br>**Funded studies**<br>Enter a statement with the following details:<br>• Initials of the authors who received each award<br>• Grant numbers awarded to each author<br>• The full name of each funder<br>• URL of each funder website<br>• Have or will the sponsors or funders play any role in the study design, data collection (when applicable) and analysis, decision to publish, or preparation of the manuscript?<br>• **NO** - Include this sentence at the end of your statement: *The funders did not and will not have a role in study design, data collection and analysis, decision to publish, or preparation of the manuscript.*<br>• **YES** - Specify the role(s) played.<br><br>* typeset	
**Competing Interests**<br><br>Use the instructions below to enter a competing interest statement for this submission. On behalf of all authors, disclose any competing interests that could be perceived to bias this work—acknowledging all financial support and any other relevant financial or non-financial competing interests.<br><br>This statement is required for submission and **will appear in the published article** if the submission is accepted. Please make sure it is accurate and that any funding sources listed in your Funding Information later in the submission form are also declared in your Financial Disclosure statement.<br><br>View published research articles from *PLOS ONE* for specific examples.	The authors have declared that no competing interests exist.

**NO authors have competing interests**<br><br>Enter: *The authors have declared that no competing interests exist.*<br><br>**Authors with competing interests**<br><br>Enter competing interest details beginning with this statement:<br><br>*I have read the journal's policy and the authors of this manuscript have the following competing interests: [insert competing interests here]*<br><br>* typeset	
**Ethics Statement**<br><br>Enter an ethics statement for this submission. This statement is required if the study involved:<br><br>• Human participants<br>• Human specimens or tissue<br>• Vertebrate animals or cephalopods<br>• Vertebrate embryos or tissues<br>• Field research<br><br>Write "N/A" if the submission does not require an ethics statement.<br><br>General guidance is provided below. Consult the submission guidelines for detailed instructions. **Make sure that all information entered here is included in the Methods section of the manuscript.**	N/A

**Format for specific study types**<br><br>**Human Subject Research (involving human participants and/or tissue)**<br>• Give the name of the institutional review board or ethics committee that approved the study<br>• Include the approval number and/or a statement indicating approval of this research<br>• Indicate the form of consent obtained (written/oral) or the reason that consent was not obtained (e.g. the data were analyzed anonymously)<br><br>**Animal Research (involving vertebrate animals, embryos or tissues)**<br>• Provide the name of the Institutional Animal Care and Use Committee (IACUC) or other relevant ethics board that reviewed the study protocol, and indicate whether they approved this research or granted a formal waiver of ethical approval<br>• Include an approval number if one was obtained<br>• If the study involved *non-human primates*, add *additional details* about animal welfare and steps taken to ameliorate suffering<br>• If anesthesia, euthanasia, or any kind of animal sacrifice is part of the study, include briefly which substances and/or methods were applied<br><br>**Field Research**<br><br>Include the following details if this study involves the collection of plant, animal, or other materials from a natural setting:<br>• Field permit number<br>• Name of the institution or relevant body that granted permission	
**Data Availability**<br><br>Authors are required to make the data underlying their research findings fully available, without restriction.<br><br>If the manuscript reports pilot or preliminary results, the data underlying those results must be made available at the time of publication. A Data Availability	N/A - No results are reported

Statement describing where the data can be found is required at submission. Your answers to this question constitute the Data Availability Statement and will be published in the article, if accepted. When legal or ethical requirements prohibit public data sharing, authors must indicate how researchers may obtain access to the data. See the PLOS Data Policy and FAQ for detailed information.<br><br>**Important:** Stating 'data available on request from the author' is not sufficient. If your data are only available upon request, select 'No' for the first question and explain your situation in the text box.<br><br>For protocols without pilot or preliminary data, authors are strongly encouraged to state how they plan to share research data from their study when it is completed or published.<br><br>If your study includes pilot data, will they be made fully available without restriction?	
**Describe where the data may be found in full sentences. If you are copying our sample text, replace any instances of XXX with the appropriate details.**<br><br>• If **no pilot data are reported** in the manuscript, please enter the following: *No datasets were generated or analysed during the current study. All relevant data from this study will be made available upon study completion.*<br>• If the manuscript reports pilot data **held in a public repository**, include URLs, accession numbers or DOIs. If this information will only be available after acceptance, indicate this by ticking the box below. For example: *All XXX files are available from the XXX database (accession number(s) XXX, XXX.).*<br>• If the manuscript reports pilot data contained **within the manuscript and/or**	Generated data will be accessible via OSF once the study is completed.

**Supporting Information files**, enter the following:<br>*All relevant data are within the manuscript and its Supporting Information files.*<br>• If neither of these applies but you are able to provide **details of access elsewhere**, with or without limitations, please do so. For example:<br><br>*Data cannot be shared publicly because of [XXX]. Data are available from the XXX Institutional Data Access / Ethics Committee (contact via XXX) for researchers who meet the criteria for access to confidential data.*<br><br>*The data underlying the results presented in the study are available from (include the name of the third party and contact information or URL).*<br>• This text is appropriate if the data are owned by a third party and authors do not have permission to share the data.<br><br>For study protocols with data management or sharing plans, authors are encouraged to briefly describe in their Data Availability Statement how data they generate will be made accessible when the study is completed. For example:<br>• Deidentified research data will be made publicly available when the study is completed and published.<br><br>* typeset	
Additional data availability information:	Tick here if your circumstances are not covered by the questions above and you need the journal's help to make your data available.

Powered by Editorial Manager® and ProduXion Manager® from Aries Systems Corporation

Manuscript Click here to access/download;Manuscript;Brain fog social media protocol_2023 (1).docx

Brain fog in chronic pain: Protocol for a discourse analysis of social media postings

Ronessa Dass, BCogSciH
School of Rehabilitation Sciences
McMaster University

Dr. Tara Packham, OTReg.(Ont.), PhD
Assistant Professor, School of Rehabilitation Sciences
McMaster University

Number of text pages: 4

Number of figures and tables: 1

Contact author: Ronessa Dass
Email: dassr5@mcmaster.ca

Introduction

Brain fog is a phenomenon that is frequently reported by persons with chronic pain (1,2) . It is commonly described as difficulties with cognition including memory impairments, attentional issues, cloudiness, and more (3–6). The term 'brain fog' was initially developed by persons with lived experiences (PWLE), primarily through online discussions (7). In the medical literature, it remains poorly understood and does not have an agreed definition (7,8). To help resolve this issue, we performed a scoping review of the medical literature to inform a working definition of brain fog (8). The definition positions brain fog as subjective state of cognitive dysfunction that varies across and within individuals, impacting participation in daily activities (8). To expand our understanding of this complex phenomenon, we will perform a discourse analysis by searching text-based postings on two social media sources, Twitter and Facebook, to explore how brain fog is described in public discourses on social media.

Discourse analyses are beneficial in understanding how the discussion around a particular topic (e.g., vocabulary used, personal anecdotes and opinions) form its societal understanding and meaning (9–12). Discourses about a particular phenomenon are communication-based actions that are created by one's experiences and that are continuously evolving to shape an overall understanding of the topic (9–11). As it establishes an understanding of a topic, a discourse may in turn impact how individuals perceive a topic and engage with it (11,13). For example, the discourse individuals see about the phenomenon of brain fog may affect how they identify with their condition, how they manage it, and the role it plays in their daily lives (11,13).

Social media platforms are a powerful tool used by stakeholders including PWLE, researchers, and health care professionals to promote awareness and understanding of different conditions (14,15). Many persons with chronic pain use social media platforms for health advocacy by sharing their experiences and receiving support from others (14). Persons with chronic pain may share strategies to help others understand their experiences, communicate with health care providers, and identify management strategies (14).

The use of social media data also has many benefits for research (15). For example, social media data is not limited by geographic boundaries full in monitoring emerging public health trends to aid in estimating attributes, causes, and potential interventions (15,16).

Though social media data may be inherently biased, and at times inaccurate, the discourse often has real life implications (14,15). Persons with chronic pain may adopt the information they see online and may be influenced to alter their beliefs about their conditions (14,15). For example, individuals may use a particular intervention they see online or may describe their condition using words from social media posts (14,15). However, exploration of the vast amount of information and available on social media may have negative consequences, such as cognitive overload (17,18). This can be problematic in the context of brain fog in which cognitive load may be limited (7). Increased cognitive load may also decrease one's ability to critically evaluate and verify the information they are receiving (17). A study investigating social media fatigue during the early stages of the COVID pandemic stated that social media fatigue decreased participants fact-checking behaviour and led to information avoidance: and this avoidance had potential negative health consequences (18). The study concluded that examining how target

populations use social media for health concerns can help researchers develop effective dissemination strategies for those groups (18). Given that brain fog is a phenomenon that is predominantly discussed in online discourse (7), a deductive analysis of social media sources is highly relevant. These results may aid in 1) understanding the causes, attributes, and potential interventions for brain fog and 2) bridging the understanding between scientific sources and natural discussions.

Therefore, the overarching objective of this study will be to explore how brain fog in chronic pain is described in public discourses on several text-based social media platforms. Our specific objectives include:

1. To explore if brain fog is described differently pre and post the COVID-19 pandemic.
2. To explore if brain fog described differently on social media across the social groups of researchers, health care professionals, and persons with lived experience
3. To synthesize how the symptoms of brain fog are described in public discourses on social media
4. To identify management strategies used by persons with brain fog or suggested by others
5. To contrast our working definition of brain fog with descriptions posted on social media

Methods

Search strategy

An initial formative search of social media databases was iteratively performed to select relevant key terms and the appropriate databases.

For the purpose of this study, text-based social media posts on Twitter and Facebook will be used as data sources. Twitter and Facebook have been selected as 1) they are text centric data bases with open access, 2) they are widely used by our social groups of interest, 3) they have the opportunity for discourse amongst users, 4) they have a wide range of data source type (e.g., text, videos, and infographics), and 5) they have robust search engines (16,19,20). Key terms including "#brainfog" have been included to ensure that the search is comprehensive and does not miss any potential sources which merit inclusion ((21); See Appendix A for full search strategy). To maintain the feasibility of the study and to achieve one of our secondary objectives addressing potential shifts in discourse after the onset of the COVID pandemic, we will be restricting our search from the years 2020-2022 and 2016-2018. The search will be run by the primary researcher (RD) and will run from new purpose-created account with no memberships in any groups or communities, to reduce any ethical concerns (22).

Types of sources

Sources that will be included

- Public posts in English
- Posts discussing brain fog in adults with a painful chronic condition (e.g., chronic pain, chronic musculoskeletal pain)

- Posts must be explicitly referring to chronic pain, or refers to chronic pain in the profile, or that discusses chronic pain in adjacent thread posts.
- Text posts about or in response to a video or image

Sources that will be excluded

- Post in a language other than English
- Brain fog related to conditions other than chronic pain (e.g., fibro fog, long COVID, chemo fog)
- Posts focused on children, as they have systematic differences in cognition (23)
- Sources from closed community support groups as these are private spaces or a response to a post from a closed community (22)
- Video or image-based posts (e.g. pictures with ALT text, memes)

<u>Data extraction</u>

All posts will be saved into a data extraction form, which will be located on a password protected device and will only be shared with researchers directly involved with the project ((22); See Appendix B for an outline of the data extraction form). The data extraction form was informed by a) Foley's concept analysis framework to support systematic elaboration of the concept of brain fog (24), b) Internet Specific Ethical Questions Framework to protect data anonymity (22), c) the International Classification of Functioning to categorize the impacts described (25), and d) Dass et al's (2023) model of brain fog (8). The data extraction form will be piloted with the first ten posts and will be modified if needed, through a discussion with both reviewers (RD and TP; (21)). Exact quotes will be stored on the data extraction form, however, we will use synthetic or paraphrased quotes in the official manuscript to protect anonymity (14,26,27). Additionally, we will also collect demographic information on the stakeholder type (PWLE, researcher, or health care professional), to protect anonymity (14,26,27). Data extraction will be completed by one primary reviewer (RD), with verification review of a random sample of 10% of the posts by a second reviewer as a trustworthiness strategy.

<u>Data analysis and presentation</u>

The data derived from this review will be used to deductively underpin both a mapping review and concept analysis. In the mapping review, we will compare and contrast 1) how brain fog is described pre and post the COVID-19 pandemic and 2) how brain fog is described across 3 different stakeholder groups: researchers, health care professionals, and persons with lived experience. In the concept analysis, we will explore the current use and meaning of brain fog, by identifying its attributes, antecedents, and potential interventions (24). Both approaches to the data will rely on deductive qualitative content analysis techniques including in-depth topical summaries, as well as frequency reports to illustrate the prevalence of each theme identified during data analysis.

Implications

As described, the results of the proposed discourse analysis will assist in improving the overall understanding chronic pain related brain fog by integrating knowledge from social discussions with existing scientific literature. It is anticipated that this will generate social impacts to 1) help healthcare professionals better understand the lived experience of chronic pain related brain fog and 2) identify areas of exploration for future research.

Understanding how chronic pain related brain fog is discussed in social discourses will help healthcare professionals identify how to adapt treatments to best support persons with lived experiences (13,15). First, the proposed study aims to inform the refinement of a definition of brain fog that incorporates information from both academic and lay discourses. This definition may help healthcare professionals identify brain fog in clinical settings. Additionally, the results may identify any common sources of misinformation or miscommunication, which may act as a barrier towards clinician-patient rapport (16,17).

Further, the results of this study will help researchers account for brain fog within their studies, by providing a definition supported by both academic sources and persons with lived experiences. The results may also help researchers develop educational resources for both healthcare professionals and persons with lived experiences to promote a better social understanding of chronic pain related brain fog. Lastly, understanding how persons with lived experiences describe their experiences with brain fog and their unique concerns, will generate avenues of future research to improve the overall understanding of this phenomenon.

Conclusion

To conclude, by incorporating an innovative and current methodology, this proposed discourse analysis of social media sources will generate nuanced insights, bridging the gap between the academic and lay community.

Funding
There is no funding dedicated to this project. The first author (RD) is funded by the Chronic Pain Centre of Excellence for Veterans with Chronic Pain for other projects.

Competing interests
There are no competing interests in this project.

References

1. Kucyi A, Davis KD. The dynamic pain connectome. Trends Neurosci [Internet]. 2015;38(2):86–95. Available from: http://dx.doi.org/10.1016/j.tins.2014.11.006
2. Robinson D. Managing brain fog due to chronic pain. Oklahoma pain doc. 2021;
3. Lucius K. Brain Fog": Exploring a Symptom Commonly Encountered in Clinical Practice". Mary Ann Liebert. 2021;27(1):1–9.
4. Ocon AJ. Caught in the thickness of brain fog: Exploring the cognitive symptoms of Chronic Fatigue Syndrome. Front Physiol. 2013;4:63.
5. Theoharis, C., Cholevas, C., Polyzoidis, K., Politis A. Long-covid syndrome-associated brain fog and chemo frog: Luteolin to the rescue. Biofactors. 2021;47(2):232–41.
6. Stefano GB, Ptacek R, Ptackova H, Martin A, Kream RM. Selective neuronal mitochondrial targeting in SARS-CoV-2 infection affects cognitive processes to induce "Brain Fog" and results in behavioral changes that favor viral survival. Med Sci Monit. 2021;27:1–4.
7. Yong E. One of long COVID's worst symptoms is also its most misunderstood. Atl [Internet]. 2022; Available from: https://www.theatlantic.com/health/archive/2022/09/long-covid-brain-fog-symptom-executive-function/671393/
8. Dass R, Kalia M, Packham T. Brain fog in chronic pain: A scoping review. Canadian Journal of Pain. 2023;7(1):1-20.
9. Kivle BMT, Espedal G. Identifying values through discourse analysis. Res values. 2022;177–87.
10. Publications S. In: Sage publications. 2002. p. 6–23.
11. Fjørtoft A-K, Oksholm T, Delmar C, Førland O, Alvsvåg2 H. Home-care nurses' distinctive work: A discourse analysis of what takes precedence in changing healthcare services. Nurs Inq. 2021;28(1):1–10.
12. Steinmann G, Bovenkamp H van de, Bont A de, Delnoij D. Redefining value: A discourse analysis on value-based health care. BMC Health Serv Res. 2020;20(862):1–13.
13. Mackenzie J, Murray E. Socially constructing healthy eating: A foucauldian discourse analysis of healthy eating information and advice. Qual Health Res. 2021;3(11):2135–2146. A call for a public health agenda for social media researcA call for a public health agenda for social media research.
14. Pagoto S, Waring ME, Xu R. A call for a public health agenda for social media research. JMIR Publ. 2019;21(12):1–22.
15. Zhou L, Zhang D, Yang C, Yu W. Harnessing social media for health information. PMC. 2019;27(1):139–51.
16. Chiauzzi E, Wicks P. Digital trespass: Ethical and terms-of-use violations by researchers accessing data from an online patient community. J Med Internet Res. 2019;21(2):1–12.
17. Islama AKMN, Laatob S, Talukderc S, Sutinenb E. Misinformation sharing and social media fatigue during COVID-19: An affordance and cognitive load perspective. Technol Forecast Soc Change. 2020;159(1):1–14.
18. Jiang S. The roles of worry, social media information overload, and social media fatigue in hindering health fact-checking. Soc Media + Soc. 2022;July(1):1–12.
19. Meta. Facebook community standards [Internet]. 2022. Available from: https://transparency.fb.com/policies/community-standards/

20. Twitter. The Twitter Rules [Internet]. Available from: https://help.twitter.com/en/rules-and-policies/twitter-rules
21. Shamseer L, Moher D, Clarke M, Ghersi D, Liberati A, Petticrew M, et al. Preferred reporting items for systematic review and meta-analysis protocols (PRISMA-P) 2015: Elaboration and explanation. BMJ. 2015;349(1):1–25.
22. Markham A, Buchanan E. Ethical decision making and internet research recommendations from the AoIR Ethics Working Committee (Version 2.0). AoIR Ethics Work Comm. 2012;2.
23. Murman DL. The impact of age on cognition. Semin Hear. 2015;36(3):111–21.
24. Foley AS, Davis AH. A guide to concept analysis. Clin Nurse Spec. 2017;31(2):70–3.
25. Organization WH. International classification of functionning checklist. World Heal Orgnanization. 2003;
26. Benton, A., Coppersmith, G. & Dredze M. Ethical research protocols for social media health research. In: Proceedings of the first workshop on ethics in natural language processing. 2017. p. 94–102.
27. Hunter, R. F., Gough, A., O'Kane, N., McKeown, G., Fitzpatrick, A., Walker, T., ... & Kee F. Ethical issues in social media research for public health. Am J Public Health. 2018;108(3):343–8.

Manuscript
Click here to access/download;Manuscript;Brain fog social media protocol_2023.docx

Brain fog in chronic pain: Protocol for a discourse analysis of social media postings

Ronessa Dass, BCogSciH
School of Rehabilitation Sciences
McMaster University

Dr. Tara Packham, OTReg.(Ont.), PhD
Assistant Professor, School of Rehabilitation Sciences
McMaster University

Number of text pages: 4

Number of figures and tables: 1

Contact author: Ronessa Dass
Email: dassr5@mcmaster.ca

Introduction

Brain fog is a phenomenon that is frequently reported by persons with chronic pain (1,2) . It is commonly described as difficulties with cognition including memory impairments, attentional issues, cloudiness, and more (3–6). The term ‘brain fog’ was initially developed by persons with lived experiences (PWLE), primarily through online discussions (7). In the medical literature, it remains poorly understood and does not have an agreed definition (7,8). To help resolve this issue, we performed a scoping review of the medical literature to inform a working definition of brain fog (8). The definition positions brain fog as subjective state of cognitive dysfunction that varies across and within individuals, impacting participation in daily activities (8). To expand our understanding of this complex phenomenon, we will perform a discourse analysis by searching text-based postings on two social media sources, Twitter and Facebook, to explore how brain fog is described in public discourses on social media.

Discourse analyses are beneficial in understanding how the discussion around a particular topic (e.g., vocabulary used, personal anecdotes and opinions) form its societal understanding and meaning (9–12). Discourses about a particular phenomenon are communication-based actions that are created by one’s experiences and that are continuously evolving to shape an overall understanding of the topic (9–11). As it establishes an understanding of a topic, a discourse may in turn impact how individuals perceive a topic and engage with it (11,13). For example, the discourse individuals see about the phenomenon of brain fog may affect how they identify with their condition, how they manage it, and the role it plays in their daily lives (11,13).

Social media platforms are a powerful tool used by stakeholders including PWLE, researchers, and health care professionals to promote awareness and understanding of different conditions (14,15). Many persons with chronic pain use social media platforms for health advocacy by sharing their experiences and receiving support from others (14). Persons with chronic pain may share strategies to help others understand their experiences, communicate with health care providers, and identify management strategies (14).

The use of social media data also has many benefits for research (15). For example, social media data is not limited by geographic boundaries full in monitoring emerging public health trends to aid in estimating attributes, causes, and potential interventions (15,16).

Though social media data may be inherently biased, and at times inaccurate, the discourse often has real life implications (14,15). Persons with chronic pain may adopt the information they see online and may be influenced to alter their beliefs about their conditions (14,15). For example, individuals may use a particular intervention they see online or may describe their condition using words from social media posts (14,15). However, exploration of the vast amount of information and available on social media may have negative consequences, such as cognitive overload (17,18). This can be problematic in the context of brain fog in which cognitive load may be limited (7). Increased cognitive load may also decrease one’s ability to critically evaluate and verify the information they are receiving (17). A study investigating social media fatigue during the early stages of the COVID pandemic stated that social media fatigue decreased participants fact-checking behaviour and led to information avoidance: and this avoidance had potential negative health consequences (18). The study concluded that examining how target

populations use social media for health concerns can help researchers develop effective dissemination strategies for those groups (18). Given that brain fog is a phenomenon that is predominantly discussed in online discourse (7), a deductive analysis of social media sources is highly relevant. These results may aid in 1) understanding the causes, attributes, and potential interventions for brain fog and 2) bridging the understanding between scientific sources and natural discussions.

Therefore, the overarching objective of this study will be to explore how brain fog in chronic pain is described in public discourses on several text-based social media platforms. Our specific objectives include:

1. To explore if brain fog is described differently pre and post the COVID-19 pandemic.
2. To explore if brain fog described differently on social media across the social groups of researchers, health care professionals, and persons with lived experience
3. To synthesize how the symptoms of brain fog are described in public discourses on social media
4. To identify management strategies used by persons with brain fog or suggested by others
5. To contrast our working definition of brain fog with descriptions posted on social media

Methods

<u>Search strategy</u>

An initial formative search of social media databases was iteratively performed to select relevant key terms and the appropriate databases.

For the purpose of this study, text-based social media posts on Twitter and Facebook will be used as data sources. Twitter and Facebook have been selected as 1) they are text centric data bases with open access, 2) they are widely used by our social groups of interest, 3) they have the opportunity for discourse amongst users, 4) they have a wide range of data source type (e.g., text, videos, and infographics), and 5) they have robust search engines (16,19,20). Search terms "#brainfog chronic pain," "brain fog chronic pain," "#brainfog chronic pain" and "brain fog #chronicpain" will be used to ensure that the search is comprehensive and does not miss any potential sources which merit inclusion ((21); See Appendix A for full search strategy). To maintain the feasibility of the study and to achieve one of our secondary objectives addressing potential shifts in discourse after the onset of the COVID pandemic, we will be restricting our search from the years 2020-2022 and 2016-2018. The search will be run by the primary researcher (RD) and will run from new purpose-created account with no memberships in any groups or communities, to reduce any ethical concerns (22). No other filters will be applied. In accordance the Internet Specific Ethical Questions Framework to protect data anonymity (22), searches will occur manually through a public search engine, therefore no private posts, pages, or groups will be searched. All searches will be conducted within the timespan of one week, to ensure feasibility as new posts generate daily on social media platform.

<u>Types of sources</u>

Sources that will be included

- Public posts in English
- Posts discussing brain fog in adults with a painful chronic condition (e.g., chronic pain, chronic musculoskeletal pain)
- Posts must be explicitly referring to chronic pain, or refers to chronic pain in the profile, or that discusses chronic pain in adjacent thread posts.
- Text posts about or in response to a video or image

Sources that will be excluded

- Post in a language other than English
- Brain fog related to conditions other than chronic pain (e.g., fibro fog, long COVID, chemo fog)
- Posts focused on children, as they have systematic differences in cognition (23)
- Sources from closed community support groups as these are private spaces or a response to a post from a closed community (22)
- Video or image-based posts (e.g. pictures with ALT text, memes)
- Retweets or duplicated tweets

Data extraction

All posts will be saved into a data extraction form, which will be located on a password protected device and will only be shared with researchers directly involved with the project ((22); See Appendix B for an outline of the data extraction form). The data extraction form was informed by a) Foley's concept analysis framework to support systematic elaboration of the concept of brain fog (24), b) Internet Specific Ethical Questions Framework to protect data anonymity (22), c) the International Classification of Functioning to categorize the impacts described (25), and d) Dass et al's (2023) model of brain fog (8). The data extraction form will be piloted with the first ten posts and will be modified if needed, through a discussion with both reviewers (RD and TP; (21)). Exact quotes will be stored on the data extraction form, however, we will use synthetic or paraphrased quotes in the official manuscript to protect anonymity (14,26,27). Additionally, we will also collect demographic information on the stakeholder type (PWLE, researcher, or health care professional; 14,26,27). We will manually label and classify stakeholders using information from the user profile. Data extraction will be completed manually by two researchers, as a trustworthiness strategy. All extracted information will be deleted post manuscript completion.

Data analysis and presentation

The data derived from this review will be used to deductively underpin both a mapping review and concept analysis. In the mapping analysis, we will compare and contrast 1) how brain fog is described pre and post the COVID-19 pandemic and 2) how brain fog is described across 3 different stakeholder groups: researchers, health care professionals, and persons with lived experience. The mapping analysis will occur through Microsoft excel. In the concept analysis, we will explore the current use and meaning of brain fog, by identifying its attributes, antecedents, and potential interventions (24). The concept analysis will occur through the qualitative analysis software, Quirkos (1). Both approaches to the data will rely on deductive qualitative content analysis techniques including in-depth topical summaries, as well as

frequency reports to illustrate the prevalence of each theme identified during data analysis. To minimize bias and improve the reliability, credibility, and quality of findings data will be independently analyzed by two researchers.

Implications

As described, the results of the proposed discourse analysis will assist in improving the overall understanding chronic pain related brain fog by integrating knowledge from social discussions with existing scientific literature. It is anticipated that this will generate social impacts to 1) help healthcare professionals better understand the lived experience of chronic pain related brain fog and 2) identify areas of exploration for future research.

Understanding how chronic pain related brain fog is discussed in social discourses will help healthcare professionals identify how to adapt treatments to best support persons with lived experiences (13,15). First, the proposed study aims to inform the refinement of a definition of brain fog that incorporates information from both academic and lay discourses. This definition may help healthcare professionals identify brain fog in clinical settings. Additionally, the results may identify any common sources of misinformation or miscommunication, which may act as a barrier towards clinician-patient rapport (16,17).

Further, the results of this study will help researchers account for brain fog within their studies, by providing a definition supported by both academic sources and persons with lived experiences. The results may also help researchers develop educational resources for both healthcare professionals and persons with lived experiences to promote a better social understanding of chronic pain related brain fog. Lastly, understanding how persons with lived experiences describe their experiences with brain fog and their unique concerns, will generate avenues of future research to improve the overall understanding of this phenomenon.

Conclusion

To conclude, by incorporating an innovative and current methodology, this proposed discourse analysis of social media sources will generate nuanced insights, bridging the gap between the academic and lay community.

Funding
There is no funding dedicated to this project. The first author (RD) is funded by the Chronic Pain Centre of Excellence for Veterans with Chronic Pain for other projects.

Competing interests
There are no competing interests in this project.

References

1. Kucyi A, Davis KD. The dynamic pain connectome. Trends Neurosci [Internet]. 2015;38(2):86–95. Available from: http://dx.doi.org/10.1016/j.tins.2014.11.006
2. Robinson D. Managing brain fog due to chronic pain. Oklahoma pain doc. 2021;
3. Lucius K. Brain Fog": Exploring a Symptom Commonly Encountered in Clinical Practice". Mary Ann Liebert. 2021;27(1):1–9.
4. Ocon AJ. Caught in the thickness of brain fog: Exploring the cognitive symptoms of Chronic Fatigue Syndrome. Front Physiol. 2013;4:63.
5. Theoharis, C., Cholevas, C., Polyzoidis, K., Politis A. Long-covid syndrome-associated brain fog and chemo frog: Luteolin to the rescue. Biofactors. 2021;47(2):232–41.
6. Stefano GB, Ptacek R, Ptackova H, Martin A, Kream RM. Selective neuronal mitochondrial targeting in SARS-CoV-2 infection affects cognitive processes to induce "Brain Fog" and results in behavioral changes that favor viral survival. Med Sci Monit. 2021;27:1–4.
7. Yong E. One of long COVID's worst symptoms is also its most misunderstood. Atl [Internet]. 2022; Available from: https://www.theatlantic.com/health/archive/2022/09/long-covid-brain-fog-symptom-executive-function/671393/
8. Dass R, Kalia M, Packham T. Brain fog in chronic pain: A scoping review. Canadian Journal of Pain. 2023;7(1):1-20.
9. Kivle BMT, Espedal G. Identifying values through discourse analysis. Res values. 2022;177–87.
10. Publications S. In: Sage publications. 2002. p. 6–23.
11. Fjørtoft A-K, Oksholm T, Delmar C, Førland O, Alvsvåg2 H. Home-care nurses' distinctive work: A discourse analysis of what takes precedence in changing healthcare services. Nurs Inq. 2021;28(1):1–10.
12. Steinmann G, Bovenkamp H van de, Bont A de, Delnoij D. Redefining value: A discourse analysis on value-based health care. BMC Health Serv Res. 2020;20(862):1–13.
13. Mackenzie J, Murray E. Socially constructing healthy eating: A foucauldian discourse analysis of healthy eating information and advice. Qual Health Res. 2021;3(11):2135–2146. A call for a public health agenda for social media researcA call for a public health agenda for social media research.
14. Pagoto S, Waring ME, Xu R. A call for a public health agenda for social media research. JMIR Publ. 2019;21(12):1–22.
15. Zhou L, Zhang D, Yang C, Yu W. Harnessing social media for health information. PMC. 2019;27(1):139–51.
16. Chiauzzi E, Wicks P. Digital trespass: Ethical and terms-of-use violations by researchers accessing data from an online patient community. J Med Internet Res. 2019;21(2):1–12.
17. Islama AKMN, Laatob S, Talukderc S, Sutinenb E. Misinformation sharing and social media fatigue during COVID-19: An affordance and cognitive load perspective. Technol Forecast Soc Change. 2020;159(1):1–14.
18. Jiang S. The roles of worry, social media information overload, and social media fatigue in hindering health fact-checking. Soc Media + Soc. 2022;July(1):1–12.
19. Meta. Facebook community standards [Internet]. 2022. Available from: https://transparency.fb.com/policies/community-standards/

20. Twitter. The Twitter Rules [Internet]. Available from: https://help.twitter.com/en/rules-and-policies/twitter-rules
21. Shamseer L, Moher D, Clarke M, Ghersi D, Liberati A, Petticrew M, et al. Preferred reporting items for systematic review and meta-analysis protocols (PRISMA-P) 2015: Elaboration and explanation. BMJ. 2015;349(1):1–25.
22. Markham A, Buchanan E. Ethical decision making and internet research recommendations from the AoIR Ethics Working Committee (Version 2.0). AoIR Ethics Work Comm. 2012;2.
23. Murman DL. The impact of age on cognition. Semin Hear. 2015;36(3):111–21.
24. Foley AS, Davis AH. A guide to concept analysis. Clin Nurse Spec. 2017;31(2):70–3.
25. Organization WH. International classification of functionning checklist. World Heal Orgnanization. 2003;
26. Benton, A., Coppersmith, G. & Dredze M. Ethical research protocols for social media health research. In: Proceedings of the first workshop on ethics in natural language processing. 2017. p. 94–102.
27. Hunter, R. F., Gough, A., O'Kane, N., McKeown, G., Fitzpatrick, A., Walker, T., ... & Kee F. Ethical issues in social media research for public health. Am J Public Health. 2018;108(3):343–8.

Click here to access/download;Figure;Social media model (1).pdf

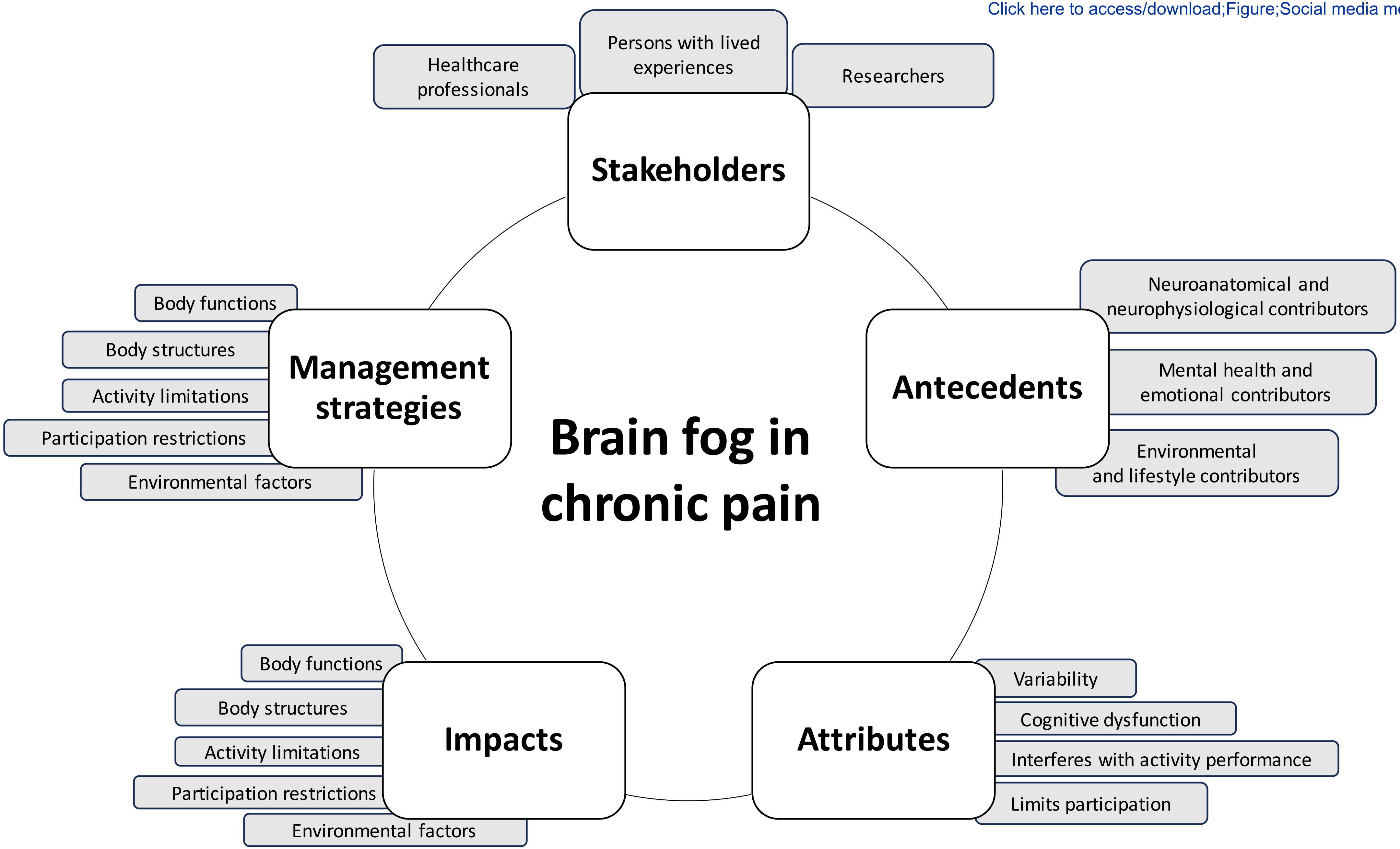

Revised Manuscript with Track Changes

Brain fog in chronic pain: Protocol for a discourse analysis of social media postings

Ronessa Dass, BCogSciH
School of Rehabilitation Sciences
McMaster University

Dr. Tara Packham, OTReg.(Ont.), PhD
Assistant Professor, School of Rehabilitation Sciences
McMaster University

Number of text pages: 4

Number of figures and tables: 1

Contact author: Ronessa Dass
Email: dassr5@mcmaster.ca

Introduction

Brain fog is a phenomenon that is frequently reported by persons with chronic pain (1,2) . It is commonly described as difficulties with cognition including memory impairments, attentional issues, cloudiness, and more (3–6). The term 'brain fog' was initially developed by persons with lived experiences (PWLE), primarily through online discussions (7). In the medical literature, it remains poorly understood and does not have an agreed definition (7,8). To help resolve this issue, we performed a scoping review of the medical literature to inform a working definition of brain fog (8). The definition positions brain fog as subjective state of cognitive dysfunction that varies across and within individuals, impacting participation in daily activities (8). To expand our understanding of this complex phenomenon, we will perform a discourse analysis by searching text-based postings on two social media sources, Twitter and Facebook, to explore how brain fog is described in public discourses on social media.

Discourse analyses are beneficial in understanding how the discussion around a particular topic (e.g., vocabulary used, personal anecdotes and opinions) form its societal understanding and meaning (9–12). Discourses about a particular phenomenon are communication-based actions that are created by one's experiences and that are continuously evolving to shape an overall understanding of the topic (9–11). As it establishes an understanding of a topic, a discourse may in turn impact how individuals perceive a topic and engage with it (11,13). For example, the discourse individuals see about the phenomenon of brain fog may affect how they identify with their condition, how they manage it, and the role it plays in their daily lives (11,13).

Social media platforms are a powerful tool used by stakeholders including PWLE, researchers, and health care professionals to promote awareness and understanding of different conditions (14,15). Many persons with chronic pain use social media platforms for health advocacy by sharing their experiences and receiving support from others (14). Persons with chronic pain may share strategies to help others understand their experiences, communicate with health care providers, and identify management strategies (14).

The use of social media data also has many benefits for research (15). For example, social media data is not limited by geographic boundaries full in monitoring emerging public health trends to aid in estimating attributes, causes, and potential interventions (15,16).

Though social media data may be inherently biased, and at times inaccurate, the discourse often has real life implications (14,15). Persons with chronic pain may adopt the information they see online and may be influenced to alter their beliefs about their conditions (14,15). For example, individuals may use a particular intervention they see online or may describe their condition using words from social media posts (14,15). However, exploration of the vast amount of information and available on social media may have negative consequences, such as cognitive overload (17,18). This can be problematic in the context of brain fog in which cognitive load may be limited (7). Increased cognitive load may also decrease one's ability to critically evaluate and verify the information they are receiving (17). A study investigating social media fatigue during the early stages of the COVID pandemic stated that social media fatigue decreased participants fact-checking behaviour and led to information avoidance: and this avoidance had potential negative health consequences (18). The study concluded that examining how target

populations use social media for health concerns can help researchers develop effective dissemination strategies for those groups (18). Given that brain fog is a phenomenon that is predominantly discussed in online discourse (7), a deductive analysis of social media sources is highly relevant. These results may aid in 1) understanding the causes, attributes, and potential interventions for brain fog and 2) bridging the understanding between scientific sources and natural discussions.

Therefore, the overarching objective of this study will be to explore how brain fog in chronic pain is described in public discourses on several text-based social media platforms. Our specific objectives include:

1. To explore if brain fog is described differently pre and post the COVID-19 pandemic.
2. To explore if brain fog described differently on social media across the social groups of researchers, health care professionals, and persons with lived experience
3. To synthesize how the symptoms of brain fog are described in public discourses on social media
4. To identify management strategies used by persons with brain fog or suggested by others
5. To contrast our working definition of brain fog with descriptions posted on social media

Methods

Search strategy

An initial formative search of social media databases was iteratively performed to select relevant key terms and the appropriate databases.

For the purpose of this study, text-based social media posts on Twitter and Facebook will be used as data sources. Twitter and Facebook have been selected as 1) they are text centric data bases with open access, 2) they are widely used by our social groups of interest, 3) they have the opportunity for discourse amongst users, 4) they have a wide range of data source type (e.g., text, videos, and infographics), and 5) they have robust search engines (16,19,20). Search terms ~~Key terms including~~ “#brainfog chronic pain,” “brain fog chronic pain,” “#brainfog chronic pain” and “brain fog #chronicpain” will be ~~have been included to~~used to ensure that the search is comprehensive and does not miss any potential sources which merit inclusion ((21); See Appendix A for full search strategy). To maintain the feasibility of the study and to achieve one of our secondary objectives addressing potential shifts in discourse after the onset of the COVID pandemic, we will be restricting our search from the years 2020-2022 and 2016-2018. The search will be run by the primary researcher (RD) and will run from new purpose-created account with no memberships in any groups or communities, to reduce any ethical concerns (22). No other filters will be applied. In accordance the Internet Specific Ethical Questions Framework to protect data anonymity (22), searches will occur manually through a public search engine, therefore no private posts, pages, or groups will be searched. All searches will be conducted within the timespan of one week, to ensure feasibility as new posts generate daily on social media platform.

Types of sources

Sources that will be included

- Public posts in English
- Posts discussing brain fog in adults with a painful chronic condition (e.g., chronic pain, chronic musculoskeletal pain)
- Posts must be explicitly referring to chronic pain, or refers to chronic pain in the profile, or that discusses chronic pain in adjacent thread posts.
- Text posts about or in response to a video or image

Sources that will be excluded

- Post in a language other than English
- Brain fog related to conditions other than chronic pain (e.g., fibro fog, long COVID, chemo fog)
- Posts focused on children, as they have systematic differences in cognition (23)
- Sources from closed community support groups as these are private spaces or a response to a post from a closed community (22)
- Video or image-based posts (e.g. pictures with ALT text, memes)
- Retweets or duplicated tweets

Formatted: Font: Font color: Auto

Data extraction

All posts will be saved into a data extraction form, which will be located on a password protected device and will only be shared with researchers directly involved with the project ((22); See Appendix B for an outline of the data extraction form). The data extraction form was informed by a) Foley's concept analysis framework to support systematic elaboration of the concept of brain fog (24), b) Internet Specific Ethical Questions Framework to protect data anonymity (22), c) the International Classification of Functioning to categorize the impacts described (25), and d) Dass et al's (2023) model of brain fog (8). The data extraction form will be piloted with the first ten posts and will be modified if needed, through a discussion with both reviewers (RD and TP; (21)). Exact quotes will be stored on the data extraction form, however, we will use synthetic or paraphrased quotes in the official manuscript to protect anonymity (14,26,27). Additionally, we will also collect demographic information on the stakeholder type (PWLE, researcher, or health care professional; l), to protect anonymity (14,26,27). We will manually label and classify stakeholders using information from the user profile. Data extraction will be completed by completed manually by two researchers, one primary reviewer (RD), with verification review of a random sample of 10% of the posts by a second reviewer as a trustworthiness strategy. All extracted information will be deleted post manuscript completion.

Formatted: Highlight

Formatted: Highlight

Data analysis and presentation

The data derived from this review will be used to deductively underpin both a mapping review and concept analysis. In the mapping analysisreview, we will compare and contrast 1) how brain fog is described pre and post the COVID-19 pandemic and 2) how brain fog is described across 3 different stakeholder groups: researchers, health care professionals, and persons with lived experience. The mapping analysis will occur through Microsoft excel. In the concept analysis, we will explore the current use and meaning of brain fog, by identifying its attributes,

antecedents, and potential interventions (24). The concept analysis will occur through the qualitative analysis software, Quirkos (1). Both approaches to the data will rely on deductive qualitative content analysis techniques including in-depth topical summaries, as well as frequency reports to illustrate the prevalence of each theme identified during data analysis. To minimize bias and improve the reliability, credibility, and quality of findings data will be independently analyzed by two researchers.

Implications

As described, the results of the proposed discourse analysis will assist in improving the overall understanding chronic pain related brain fog by integrating knowledge from social discussions with existing scientific literature. It is anticipated that this will generate social impacts to 1) help healthcare professionals better understand the lived experience of chronic pain related brain fog and 2) identify areas of exploration for future research.

Understanding how chronic pain related brain fog is discussed in social discourses will help healthcare professionals identify how to adapt treatments to best support persons with lived experiences (13,15). First, the proposed study aims to inform the refinement of a definition of brain fog that incorporates information from both academic and lay discourses. This definition may help healthcare professionals identify brain fog in clinical settings. Additionally, the results may identify any common sources of misinformation or miscommunication, which may act as a barrier towards clinician-patient rapport (16,17).

Further, the results of this study will help researchers account for brain fog within their studies, by providing a definition supported by both academic sources and persons with lived experiences. The results may also help researchers develop educational resources for both healthcare professionals and persons with lived experiences to promote a better social understanding of chronic pain related brain fog. Lastly, understanding how persons with lived experiences describe their experiences with brain fog and their unique concerns, will generate avenues of future research to improve the overall understanding of this phenomenon.

Conclusion

To conclude, by incorporating an innovative and current methodology, this proposed discourse analysis of social media sources will generate nuanced insights, bridging the gap between the academic and lay community.

Funding
There is no funding dedicated to this project. The first author (RD) is funded by the Chronic Pain Centre of Excellence for Veterans with Chronic Pain for other projects.

Competing interests
There are no competing interests in this project.

References

1. Kucyi A, Davis KD. The dynamic pain connectome. Trends Neurosci [Internet]. 2015;38(2):86–95. Available from: http://dx.doi.org/10.1016/j.tins.2014.11.006
2. Robinson D. Managing brain fog due to chronic pain. Oklahoma pain doc. 2021;
3. Lucius K. Brain Fog": Exploring a Symptom Commonly Encountered in Clinical Practice". Mary Ann Liebert. 2021;27(1):1–9.
4. Ocon AJ. Caught in the thickness of brain fog: Exploring the cognitive symptoms of Chronic Fatigue Syndrome. Front Physiol. 2013;4:63.
5. Theoharis, C., Cholevas, C., Polyzoidis, K., Politis A. Long-covid syndrome-associated brain fog and chemo frog: Luteolin to the rescue. Biofactors. 2021;47(2):232–41.
6. Stefano GB, Ptacek R, Ptackova H, Martin A, Kream RM. Selective neuronal mitochondrial targeting in SARS-CoV-2 infection affects cognitive processes to induce "Brain Fog" and results in behavioral changes that favor viral survival. Med Sci Monit. 2021;27:1–4.
7. Yong E. One of long COVID's worst symptoms is also its most misunderstood. Atl [Internet]. 2022; Available from: https://www.theatlantic.com/health/archive/2022/09/long-covid-brain-fog-symptom-executive-function/671393/
8. Dass R, Kalia M, Packham T. Brain fog in chronic pain: A scoping review. Canadian Journal of Pain. 2023;7(1):1-20.
9. Kivle BMT, Espedal G. Identifying values through discourse analysis. Res values. 2022;177–87.
10. Publications S. In: Sage publications. 2002. p. 6–23.
11. Fjørtoft A-K, Oksholm T, Delmar C, Førland O, Alvsvåg2 H. Home-care nurses' distinctive work: A discourse analysis of what takes precedence in changing healthcare services. Nurs Inq. 2021;28(1):1–10.
12. Steinmann G, Bovenkamp H van de, Bont A de, Delnoij D. Redefining value: A discourse analysis on value-based health care. BMC Health Serv Res. 2020;20(862):1–13.
13. Mackenzie J, Murray E. Socially constructing healthy eating: A foucauldian discourse analysis of healthy eating information and advice. Qual Health Res. 2021;3(11):2135–2146. A call for a public health agenda for social media researcA call for a public health agenda for social media research.
14. Pagoto S, Waring ME, Xu R. A call for a public health agenda for social media research. JMIR Publ. 2019;21(12):1–22.
15. Zhou L, Zhang D, Yang C, Yu W. Harnessing social media for health information. PMC. 2019;27(1):139–51.
16. Chiauzzi E, Wicks P. Digital trespass: Ethical and terms-of-use violations by researchers accessing data from an online patient community. J Med Internet Res. 2019;21(2):1–12.
17. Islama AKMN, Laatob S, Talukderc S, Sutinenb E. Misinformation sharing and social media fatigue during COVID-19: An affordance and cognitive load perspective. Technol Forecast Soc Change. 2020;159(1):1–14.
18. Jiang S. The roles of worry, social media information overload, and social media fatigue in hindering health fact-checking. Soc Media + Soc. 2022;July(1):1–12.
19. Meta. Facebook community standards [Internet]. 2022. Available from: https://transparency.fb.com/policies/community-standards/

20. Twitter. The Twitter Rules [Internet]. Available from: https://help.twitter.com/en/rules-and-policies/twitter-rules
21. Shamseer L, Moher D, Clarke M, Ghersi D, Liberati A, Petticrew M, et al. Preferred reporting items for systematic review and meta-analysis protocols (PRISMA-P) 2015: Elaboration and explanation. BMJ. 2015;349(1):1–25.
22. Markham A, Buchanan E. Ethical decision making and internet research recommendations from the AoIR Ethics Working Committee (Version 2.0). AoIR Ethics Work Comm. 2012;2.
23. Murman DL. The impact of age on cognition. Semin Hear. 2015;36(3):111–21.
24. Foley AS, Davis AH. A guide to concept analysis. Clin Nurse Spec. 2017;31(2):70–3.
25. Organization WH. International classification of functionning checklist. World Heal Orgnanization. 2003;
26. Benton, A., Coppersmith, G. & Dredze M. Ethical research protocols for social media health research. In: Proceedings of the first workshop on ethics in natural language processing. 2017. p. 94–102.
27. Hunter, R. F., Gough, A., O'Kane, N., McKeown, G., Fitzpatrick, A., Walker, T., ... & Kee F. Ethical issues in social media research for public health. Am J Public Health. 2018;108(3):343–8.

December 9th, 2023

Dear Plos ONE,

Thank you to the reviewers for their insightful feedback. We have done our best to address all comments point by point.

Reviewer #1: Much of the information found on social media and forums may not be based on scientific evidence, making it difficult to differentiate between anecdotal reports and verified research findings. Therefore, I am not satisfied this is a viable methodology.

Thank you for highlighting your concerns. We acknowledge the limitations of social media posts in our introduction and will be sure to reiterate this point once we present our findings.

"Though social media data may be inherently biased, and at times inaccurate, the discourse often has real life implications (14,15). Persons with chronic pain may adopt the information they see online and may be influenced to alter their beliefs about their conditions (14,15)."

However, our objective for this study is not to describe what has been stated in the academic literature, but rather we are interested in understanding the discourse of brain fog by persons with lived experiences. This is particularly important considering that the term brain fog was first derived through online discussions of persons with lived experiences. In our final manuscript, we will report how people describe this phenomenon. This information will be used to provide recommendations for future areas of research.

Reviewer #2: This manuscript requires a major review.
Please find below what needs to be included in the Protocol for it to be publishable.

Data collection:
You need to list
• Whether you are accessing the Twitter and Facebook public stream?/How do you access Facebook data if profiles are closed/Will go to specific brain fog sites, will you become a member of these sites?/What package you are using to access Twitter stream (.i.e. python)?

In accordance with published ethical guidance, we have specified this information as follows:

In accordance the Internet Specific Ethical Questions Framework to protect data anonymity (22), searches will occur through a public search engine, therefore no private posts, pages, or groups will be searched.

We will acknowledge this approach as a limitation in the resulting manuscript, as we may indeed miss relevant material in closed Facebook groups.

• What is the anticipate data for collection period?/Data collection duration? How many days/wks/months will you be collecting data

We started data collection after submitting our protocol. Thus, data collection occurred in October 2023 within the span of one week. Data was collected within one week to ensure feasibility as new posts generate daily on social media platform. We have specified this information at the bottom of the search strategy paragraph.

• What are the explicit search terms are going to be used?

We have specified the search terms "#brainfog chronic pain," "brain fog chronic pain," "#brainfog chronic pain" and "brain fog #chronicpain"

• Will English language filters be applied?
No language filters will be applied, we have added this detail in the search strategy.

• Will you exclude retweets to maintain originality

Thank you for highlighting this point, we have added it to our exclusion criteria.

• Will keep information on the tweet/fb post data attribution (full-length tweet text, tweet ID, creation time, and Twitter user information)?

All extracted potential identifiers will be deleted post manuscript completion, this information has been added at the end of the data extraction paragraph.

Annotation:
• Will you train classifiers to distinguish between personal and health-care related tweets/• Will you manually label all tweets?/ Will you use the content of the tweet's full text for classification or just part of?/Will you preprocess the text of the tweets by normalizing all URLs to one consistent string, removing special characters and English part of speech, converting all of the text to lowercase, and lemmatization to remove noise?/Will you look at the top 1000 occurring terms (excluding common English stop words) and manually checked if the terms were relevant to health; wellness; diseases; side effects; conditions; body parts; and/or references to other substances against standard English, medical, and slang dictionaries?

We will not be using artificial intelligence for this study, thus no classifiers or preprocessing. All searching and extraction will occur manually. We have specified this detail throughout the protocol.

Analysis:

• What tool will you use to undertake qualitative content analysis?

The mapping analysis will occur on excel and the concept analysis will be done using the qualitative software, Quirkos. This detail has been added in the data analysis paragraph on page 4.

• For transparency and rigor in qualitative research, it's conventional and widely recommended to have two or more individuals independently analyse the data. This practice serves multiple valuable purposes: Minimizing Bias: When two or more researchers independently analyse the same qualitative data, it helps mitigate individual biases. Each analyst brings their unique perspective and experiences, and by having multiple analysts, you reduce the risk of one person's biases significantly influencing the interpretation of the data. Enhancing Reliability: Independent analysis by multiple individuals improves the reliability of the findings. It allows for the assessment of inter-coder reliability or inter-rater reliability, which measures the degree of agreement among coders or analysts. A high level of agreement suggests greater confidence in the validity of the findings. Quality Assurance: The collaborative approach helps in identifying and resolving discrepancies or disagreements in coding and interpretation. This iterative process can lead to more robust and well-supported findings. Richer Insights: Different analysts may notice unique patterns, themes, or nuances within the data. Multiple perspectives can lead to a deeper and more comprehensive understanding of the qualitative material. Enhancing Credibility: In qualitative research, demonstrating rigor and transparency is crucial for establishing credibility and trustworthiness. Engaging multiple analysts and documenting their consensus-building process can enhance the credibility of your research findings. You need to include all the above in your protocol.

Thank you for highlighting this! We have stated this at the end of the data analysis paragraph:

To minimize bias and improve the reliability, credibility, and quality of findings data will be independently analyzed by two researchers.
