# Supplementary material for: Brain fog in chronic pain: Protocol for a discourse analysis of social media postings
Source: PLoS One. 2024 May 8;19(5):e0302443. doi: 10.1371/journal.pone.0302443 (PMC11078377; doi:10.1371/journal.pone.0302443)
Supplement: Attachment — Submitted filename: Response to reviewers.docx [file pone.0302443.s004.docx]

Thank you to the reviewers for taking the time to review this article and provide thoughtful feedback. For simplicity, we have responded to the comment requiring additional information.

4. Have the authors described where all data underlying the findings will be made available when the study is complete?

The [PLOS Data policy](https://journals.plos.org/plosone/s/materials-and-software-sharing) requires authors to make all data underlying the findings described in their manuscript fully available without restriction, with rare exception, at the time of publication. The data should be provided as part of the manuscript or its supporting information, or deposited to a public repository. For example, in addition to summary statistics, the data points behind means, medians and variance measures should be available. If there are restrictions on publicly sharing data—e.g. participant privacy or use of data from a third party—those must be specified.

Reviewer #2: No

Thank you for highlighting this! Since we are using sources from social media, underlying findings will not be made available to maintain the anonymity of sources (22). We have specified this at the end of the data extraction section.

Thank you for your time and please feel free to let us know if you have additional questions.
